# Thermally-Activated Shape Memory Behavior of Biodegradable Blends Based on Plasticized PLA and Thermoplastic Starch

**DOI:** 10.3390/polym16081107

**Published:** 2024-04-16

**Authors:** Valentina Sessini, Valentina Salaris, Victor Oliver-Cuenca, Agnieszka Tercjak, Stefano Fiori, Daniel López, José M. Kenny, Laura Peponi

**Affiliations:** 1 Instituto de Ciencia y Tecnología de Polímeros, ICTP-CSIC, Calle Juan de la Cierva 3, 28006 Madrid, Spain; 2 Department of Organic and Inorganic Chemistry, Alcalá University, 28871 Alcalá de Henares, Spain; 3 Group Materials + Technologies (GMT), Department of Chemical and Environmental Engineering, Polytechnic School, University of the Basque Country (UPV/EHU), Plaza Europa 1, 20018 Donostia-San Sebastián, Spain; 4 Condensia Química SA, R&D Department, C/La Cierva 8, 08184 Barcelona, Spain; 5 Department of Civil and Environmental Engineering, University of Perugia, 05100 Terni, Italy

**Keywords:** shape memory, PLA, TPS, plasticizers, polymer blends, compatibilization, biodegradable polymers

## Abstract

Biodegradable blends based on plasticized poly(lactic acid) PLA and thermoplastic starch (TPS) have been obtained. The influence of the PLA plasticizer as a compatibility agent has been studied by using two different plasticizers such as neat oligomeric lactic acid (OLA) and functionalized with maleic acid (mOLA). In particular, the morphological, thermal, and mechanical properties have been studied as well as the shape memory ability of the melt-processed materials. Therefore, the influence of the interaction between different plasticizers and the PLA matrix as well as the compatibility between the two polymeric phases on the thermally-activated shape memory properties have been studied. It is very interesting to use the same additive able to act as both plasticizer and compatibilizer, decreasing the glass transition temperature of PLA to a temperature close to the physiological one, obtaining a material suitable for potential biomedical applications. In particular, we obtain that OLA-plasticized blend (oPLA/TPS) show very good thermally-activated capability at 45 °C and 50% deformation, while the blend obtained by using maleic OLA (moPLA/TPS) did not show shape memory behavior at 45 °C and 50% deformation. This fact is due to their morphological changes and the loss of two well-distinguished phases, one acting as fixed phase and the other one acting as switching phase to typically obtain shape memory response. Therefore, the thermally-activated shape memory results show that it is very important to make a balance between plasticizer and compatibilizer, considering the need of two well-established phases to obtain shape memory response.

## 1. Introduction

In the last decades, environmental, economic, and safety challenges have focused the attention of scientists and companies to produce new biodegradable materials able to partially or totally substitute petrochemical-based plastics [1]. In this context, one of the most promising biodegradable polymer in the market, also produced by renewable resources, is the poly(lactic acid) (PLA) [2,3], an aliphatic thermoplastic polyester with several advantages such as biodegradability, biocompatibility, and compostability which promote its use in many applications from commodities to engineering plastics [4,5,6]. However, the mechanical response of PLA is low due to its high brittleness, low stretchability, and relatively poor impact strength which can limit its use in specific areas [7,8]. Many strategies have been explored in both academic and industrial fields to improve the mechanical properties of PLA and enhance its performances, such as copolymerization [9], addition of additives [10], blending with other polymers [11] as well as by the incorporation of nanofillers [12]. As expected, from the industrial point of view, polymer blending and the addition of additives can be considered as the most effective and cost-efficient methods to enhance PLA performances and use it in different applications where higher toughness is required [4,13]. Therefore, with the scope of preserving the sustainability of the final material, increasing attention has been given to the use of bio-based and renewable polymers and additives to obtain complete biodegradable and bio-based blends with improved mechanical and thermal properties with respect to the neat PLA. 

In this regard, among bio-based polymers we can find starch, a biodegradable and biocompatible polysaccharide derived from renewable resources such as potatoes, peas, maize, etc., composed of the sequence α-D-glucose linked with each other through (1 → 4) glycosidic bonds. Two different structures are characteristic for starch, amylose which is linear and a branched one called amylopectin [14]. Starch can be used as nanofillers in the form of starch nanocrystals, preserving its native crystalline form or as a matrix, in the form of thermoplastic matrix when processed with water and/or plasticizer, destructuring its granules [15]. In the recent years, blends of PLA and thermoplastic starch (TPS) have been studied as environmentally friendly materials of great interest for food packaging and biomedical applications [16,17,18]. However, the major drawback of processing PLA blends through simple physical blending is their immiscibility, poor compatibility, and the weak interfacial bonding of the blend components [19,20,21]. In particular, PLA and TPS are immiscible due to the incompatibility between the hydrophobic nature of PLA and hydrophilic nature of starch. However, the addition of additives such as compatibilizer, plasticizer, coupling agent, etc., is used to reduce the interfacial tension and therefore improve the mechanical performances of the blends [13]. Nowadays, the effect of compatibilizers and plasticizers on the morphology and mechanical properties of PLA/TPS blends using different additives have been studied demonstrating that more homogeneous morphology improved the mechanical properties and thermal stability of plasticized PLA/TPS blends [22,23,24]. 

Between the additives, oligomeric lactic acid (OLA) is proposed as a renewable alternative to common plasticizers for PLA, taking advantage of their similar chemical structure that ensures high miscibility [25]. In 2001, Martin and Averous [26] reported that 20 wt.% of OLA with respect to neat PLA was enough to efficiently improve the mechanical properties of PLA and result in a decrease of about 20 °C in its glass transition temperature (T_g_). Later, Burgos et al. [27] found that amount of OLA higher than 20 wt.% can be incorporated into PLA obtaining more ductile homogeneous materials. However, they reported that PLA with 20 wt.% was the more stable formulation maintaining constant its thermal, mechanical, and oxygen barrier properties for at least 90 days at fixed conditions. Leones et al. [28] reported that electrospun PLA-OLA fibers mats, with 20 wt.% OLA, show shape memory behavior at 40 °C and 45 °C, and Sonseca et al. [29] confirmed that extruded PLA-OLA formulation with the same content of OLA also show shape-memory at a temperature close to the physiological one. This point is very important, taking into account that one of the most promising application fields of shape memory biopolymers is the biomedical sector [30]. In particular, shape memory materials are smart materials able to respond to an external stimulus such as temperature, humidity, or light, recovering their original shape from a temporary shape previously programmed. These materials are composed of two different phases, a permanent one, responsible for recovering the original shape during the recovery stage and the switching one responsible for fixing the temporary shape during the programming stage [30]. For thermally-activated shape memory polymers, the switching phase is activated at a specific transition temperature (T_trans_) that generally is associated with its T_g_ or its melting temperature (T_m_) [30]. 

Nowadays, the use of PLA-based materials with shape memory properties in biomedical applications is still a challenge due to different reasons that limit the use of these materials in real applications [31]. The shape memory effect in PLA is calculated for deformation of about 50% reaching maximum values of programming strains of about 150% [32]. Moreover, the T_trans_ of PLA-based materials is related in general with its T_g_ that is around 60 °C. For this reason, when related to biomedical applications, it is highly desirable to have the T_trans_ slightly above body temperature to safely activate the shape memory capability avoiding the potential tissue damage due to a too high temperature. In this regard, in this work, with the main objective of increasing the flexibility of PLA without losing its shape memory effect at a temperature close to the physiological one, blends of plasticized PLA and TPS have been processed using a one-step method for potential application as resorbable implants. In particular, two lactic acid oligomers (OLA8), neat one (OLA8) and functionalized with maleic acid (mOLA8), were used to modulate physical and mechanical properties of PLA and its blends with 40% of TPS. An interesting approach is the use of an additive that could be used as both plasticizer and compatibilizer for the same blend. Mechanical, thermal, and morphological properties were studied. However, the attention was focused on their thermally-activated shape memory capability as well as on the compatibilizing effect of both plasticizers on the PLS/TPS blends, both being effects strongly correlated within themselves. 

## 2. Materials 

Poly(lactic acid) pellets (PLA3052) were supplied by NatureWork^®^ (NatureWorks LLC, Minnetonka, MN, USA). Both PLA plasticizers, GLYPLAST OLA8 (OLA8) and GLYPLAST OLA 8 MALEATE (mOLA8), were kindly provided by Condensia Quimica (Barcelona, Spain) with a Mn of about 1100 g/mol. Native pea starch was obtained from Cosucra groupe Warcoing SA, (Warcoing, Belgium), with a dry content of 85 wt.%, including 60.7 wt.% amylopectin, 35.7 wt.% amylose, 3.4 wt.% fiber, and 0.24 wt.% protein, as determined by colorimetric methods and Prosky and DUMAS methods. Starch was used as received. Glycerol (purity 97%) was purchased from VWR International (Radnor, PA, USA) and was used as starch plasticizer.

## 3. Methods

### 3.1. Preparation of Plasticized PLA

Two different plasticizers, OLA8 and mOLA8 have been used in order to process plasticized PLA. Since PLA is very hygroscopic, PLA pellets, OLA8, and mOLA8 were previously dried in a vacuum oven at 60 °C, overnight. The blends were obtained by mixing PLA pellets and 20 wt.% of plasticizer by a microextruder equipped with twin conical corotating screws (MiniLab Haake Rheomex CTW5, Thermo Scientific (Waltham, MA, USA) with a capacity of 7 cm^3^. A screw rotation rate of 100 rpm, temperature of 160 °C, and residence time of 3 min were used. Therefore, two plasticized PLA-based materials were obtained and named oPLA and moPLA when OLA8 or mOLA8 was used, respectively. 

### 3.2. PLA/TPS Blends Preparation

Two different plasticized PLA/TPS blends have been processed and characterized. First, the thermo-mechanical destructuration of native starch granules with liquid glycerol and distilled water (in the wt.% ratio of 100:25:20) was performed in a Brabender^®^ internal kneader (for 3 min at 110 °C with a rotor speed of 100 rpm) in order to obtain thermoplastic starch, as reported previously [33]. Once TPS was obtained, it was melt-blended with both PLA and the plasticizer, in one step, with a twin-screw DSM microcompounder for 3 min at 160 °C with a screw speed of 100 rpm. Therefore, two blends with 40 wt.% of TPS, with respect to PLA, were obtained and named oPLA/TPS and moPLA/TPS when oPLA and moPLA were used, respectively. The amount of plasticizer with respect to PLA was maintained constant at 20 wt.% for all the formulations, while the TPS amount was calculated at 40% with respect to the total amount of PLA and plasticizer. This choice is based on the importance of considering plasticized PLA as the base material for the thermally-activated shape memory response at a physiological temperature. Moreover, as reported in the literature, blends with 40% of TPS can be considered thermally and mechanically stable and good [34]. 

## 4. Characterization

Differential scanning calorimetry (DSC) experiments were carried out in a Mettler Toledo DSC822e instrument, under nitrogen flow (30 mL/min). The typical sample weight was around 10 mg. The cycle program consisted of a first heating stage from −90 to 180 °C at a rate of 10 °C min^−1^, followed by cooling to −90 °C and subsequent heating up to 180 °C at 10 °C min^−1^. The T_g_ was calculated from the first heating and was taken at the mid-point of heat capacity changes. The melting temperature (T_m_) and cold crystallization temperature (T_cc_) were obtained from the second heating, and the degree of crystallinity (χ_c_) was determined by using Equation (1).

(1) $$\chi_{c}=100\times \left[\frac{∆H_{m}-∆H_{\mathrm{cc}}}{{∆H_{m}^{0}}^{{}^{\circ}}}\right]\frac{1}{1-m_{f}}$$

where ΔH_m_ is the enthalpy of fusion, ΔH_cc_ is the enthalpy of cool crystallization, is the enthalpy of fusion of a 100% crystalline PLA, taken as 93 J/g, and 1 − m_f_ is the weight fraction of PLA in the sample [35].

Dynamic Mechanical Thermal Analysis (DMTA) of the samples was carried out using a DMA Q800 from TA Instrument in film tension mode with an amplitude of 5 μm, a frequency of 1 Hz, a force track of 125%, and a heating rate of 2 °C·min^−1^. Samples subjected to DMA were cut from compression-molded thin films into regular specimens of approximately 20 mm × 4 mm × 0.50 mm.

Thermogravimetric analysis (TGA) was carried out using a TA-TGA Q500 thermal analyzer. The different materials were analyzed by dynamic mode using about 10 milligrams of sample from room temperature to 800 °C at 10 °C min^−1^ under nitrogen atmosphere with a flow of 60 mL min^−1^. Temperatures at the maximum degradation rate (T_max_) were calculated from the first derivative of the TGA curves (DTG).

Mechanical properties were determined using an Instron Universal Testing Machine at a strain rate of 50 mm min^−1^. Tensile test measurements were performed on five dog-bone specimens with a width of 2 mm, thickness of 0.50 mm, and leaving an initial length between the clamps of 20 mm. From these experiments were obtained the Young Modulus, as the slope of the curve between 0% and 2% deformation, the elongation at break, and the maximum stress were reached.

SEM micrographs of the cryo-fracture surface of neat PLA and plasticized PLA were obtained by Scanning Electron Microscopy (SEM PHILIPS XL30 with a tungsten filament) in order to study their morphology. The polymer samples were frozen using liquid N_2_ and then cryo-fractured. All the samples were gold/palladium coated by an automatic sputter-coated Polaron SC7640. The investigated blends were analyzed also by Atomic Force Microscopy (AFM) operating in tapping mode with a scanning probe microscope (Icon form Bruker with Nanoscope V controller). All blends were cut using an ultra-microtome Leica Ultracut R with a diamond blade. Height and phase images were obtained under ambient conditions with typical scan speed of 0.5–1 line/s using a scan head with a maximum range of 16 μm × 16 μm. Surface roughness (the roughness average (R_a_) and root mean square roughness (R_q_)) of each investigated system was calculated using 5 μm × 5 μm AFM height image.

Samples for the thermally-activated shape memory studies were cut from compression-molded thin films into rectangular specimens of approximately 20 mm × 4 mm × 0.60 mm. All thermo-mechanical cycles were carried out using a stress-controlled DMA Q800 from TA Instruments in film tension mode. The samples were heated at the T_trans_ (for neat PLA the T_trans_ was 75 °C while for the rest of the materials, it was 45 °C) for 5 min and stretched until 50% by applying a constant deformation stress. They were then quenched at the fixing temperature, T_fix,_ (neat PLA, T_fix_ = 25 °C; rest of materials T_fix_ = 0 °C) under the same constant stress. The temporary shape, as characterized by an elongation of Ɛ_m_, was recovered after releasing the stress, and the permanent shape, characterized by an elongation of Ɛ_p_, was recovered upon heating (2 °C·min^−1^) to T_trans_. 

Therefore, to get a quantitative estimation of the shape memory properties of the material, the strain fixity ratio (R_f_), and the strain recovery ratio (R_r_) have been calculated [35]. In particular, R_r_, the ability to recover the initial shape, was taken as the ratio of the recovered strain to the total strain, as given by the following equation:

(2) $$R_{r}\left(N\right)=\frac{\left(\varepsilon_{m}-\varepsilon_{p}\left(N\right)\right)}{\varepsilon_{m}-\varepsilon_{p}\left(N-1\right)}\times 100\%$$

R_f_, the ability to fix the temporary shape, is the amplitude ratio of the fixed strain to the total strain, as presented by Equation (3):

(3) $$R_{f}\left(N\right)=\frac{\varepsilon_{u}\left(N\right)}{\varepsilon_{m}}\times 100\%$$

where Ɛ_m_ is the deformed strain, Ɛ_u_ the fixed strain, Ɛ_p_ the recovered strain, and N is the number of cycles.

## 5. Results and Discussion

The effect of two different plasticizers on the thermal and mechanical properties of PLA-based blends was first studied to evaluate their miscibility and their effects as plasticizers for PLA, and as compatibilizers for PLA/TPS blends with the ultimate scope to study their thermally-activated shape memory response. Based on previous results, we fixed the amount of plasticizer at 20 wt.% with respect to PLA [27,36], considering that this amount is enough to decrease T_g_ of PLA to a temperature close to the physiological one, with the purpose of obtaining materials with a shape memory response suitable for biomedical applications avoiding migration phenomena [27]. The preparation of blends with a composition of 40 wt.% of TPS was chosen following previous reports by Nazrin et al. [34]. 

**Figure 1:**
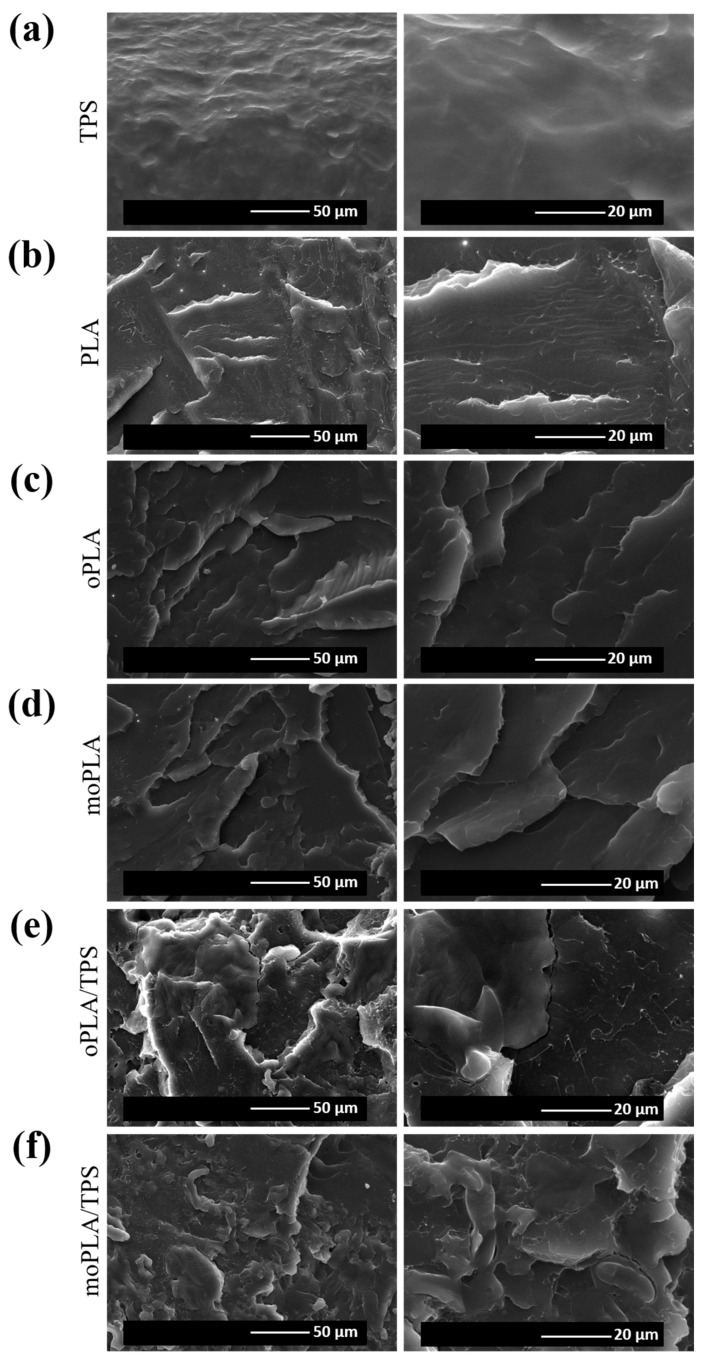
SEM images of neat TPS (**a**) PLA (**b**), plasticized PLAs (**c**,**d**), and plasticized PLA/TPS blends (**e**,**f**).

**Figure 2:**
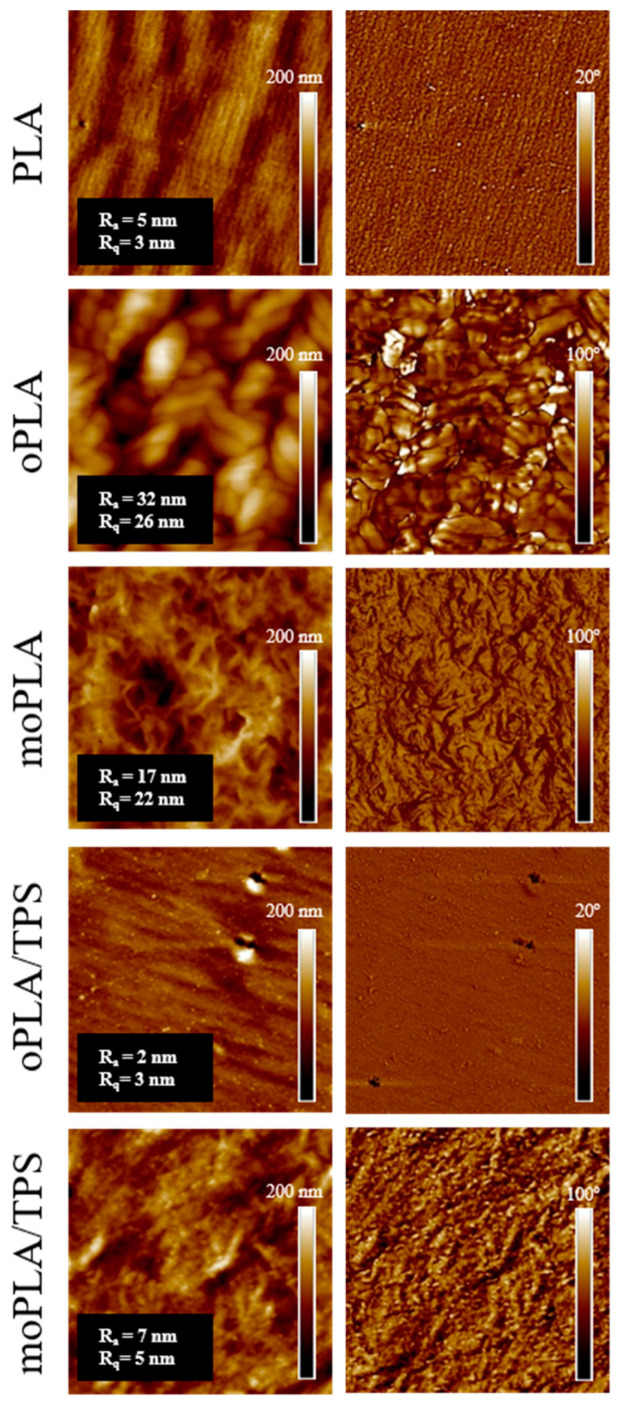
Cross-section AFM height (**left**) and phase (**right**) images (3 μm × 3 μm) of neat PLA, plasticized PLAs, and PLA/TPS blends. R_a_ and R_q_ values are given in the inset of each AFM height image.

In order to understand the properties of the processed blends, it is necessary to study their morphology. Figure 1 shows the SEM images of cryo-fractured surfaces of the samples corresponding to neat PLA, plasticized PLA with both OLA8 and mOLA8, and their respective blends obtained by adding 40 wt.% of TPS with respect to the plasticized PLA. Therefore, it is easy to note that PLA showed the typical brittle fracture with a smooth fracture surface due to its very low plastic deformation. In the case of oPLA and moPLA, no evidence of phase separation was detected. Indeed, plasticized PLAs/TPS blends showed different roughness with respect to neat PLA, also confirmed by further AFM analysis (Figure 2). This fact can be due to the difference in the hydrophilic nature of PLA and TPS phases. However, from the SEM analysis, moPLA/TPS seems to present a better dispersion of the TPS phase obtaining a more homogeneous material. This is possibly due to the improvement in interfacial adhesion between the two phases thanks to the compatibilizer effect of the plasticizer mOLA8. 

However, the effect of the addition of both OLA8 and mOLA8 on the morphology of PLA/TPS blend was deeply studied by AFM. The obtained AFM height and phase images are shown in Figure 2. The cross-section AFM phase image of PLA indicated its homogeneous morphology, which also confirmed the very low surface roughness of PLA. The addition of OLA8 and mOLA8 into PLA resulted in changes in the morphology observed for PLA. In the case of oPLA, the regular crystalline-like structure with separated domains of around 200 nm was detected while moPLA showed a different regular structure with small spherulites of the size of 400 ± 50 nm. The surface roughness of oPLA was eight times higher than the surface roughness of PLA and moPLA was four times higher, suggesting that both OLA8 and mOLA8 are good plasticizers for PLA.

In the case of PLA/TPS blends, the addition of OLA8 led to the regular structure which was very similar to the morphology of PLA. The only difference was the presence of small-in-size (~20 nm) spherical domains. The moPLA/TPS blend demonstrates still very regular spherulites-like structures well detected for moPLA with clearly visible small-in-size (~35 nm) spherical domains.

**Figure 3:**
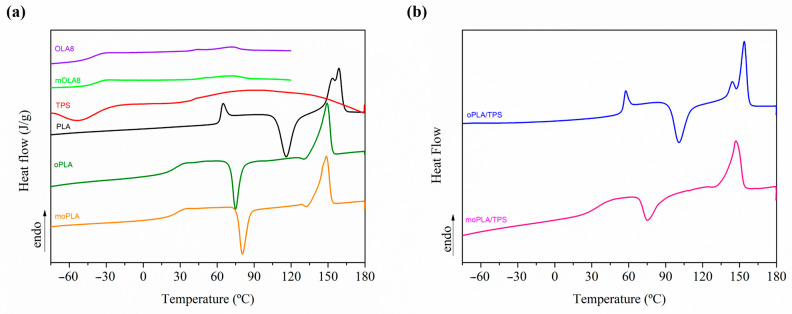
First heating scan of neat materials and plasticized PLAs (**a**) and their blends with TPS (**b**).

Figure 3 shows the DSC thermograms reporting the first heating scan for the neat materials and plasticized PLA on the left, and plasticized PLA/TPS blends on the right. The first heating scan is taken into account for the thermally activated shape memory study, considering that no further thermal treatments are applied to the samples before being tested by thermo-mechanical cycles. Therefore, from the DSC analysis, we can point out that both plasticized PLA-based materials, with OLA8 and mOLA8, showed a single T_g_, indicating the absence of macroscopic phase separation, confirming the morphological analysis, and a consequent good miscibility between the components. As expected, the incorporation of the plasticizer induced an increment in the free volume between PLA chains, consequently increasing the chain mobility which resulted in a reduction in T_g_ values for plasticized PLA-based materials in comparison with neat PLA. In particular, for plasticized PLA-based materials a T_g_ of about 25 °C is obtained, an increase of about 10 °C, when mixed with TPS. Moreover, only oPLA shows a quite high degree of crystallinity (28%) and moPLA presents a quite small degree of crystallinity (6%), the other samples being almost amorphous. However, when mixed with TPS, the degree of crystallinity of oPLA/TPS decreases up to 5%, a decrease of about 80%. For moPLA, the degree of crystallinity is quite low (6%) however, when mixed with TPS, the degree of crystallinity increases up to 20%, confirming the crystalline structure obtained by AFM.

The behavior observed when mOLA8 was used as plasticizer could be attributed to the presence of a better dispersed and compatibilized TPS phase into the PLA matrix. 

**Figure 4:**
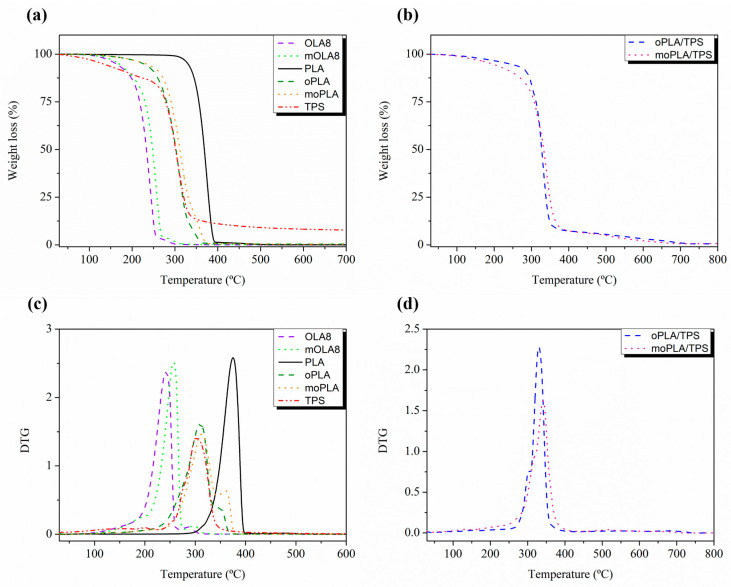
TGA of TPS, PLA, plasticizers and plasticized PLA (**a**) and blends with TPS (**b**) and DTG of TPS, PLA, plasticizers and plasticized PLA (**c**) and blends with TPS (**d**).

Moreover, the thermal stability of the materials was studied by TGA. Figure 4 shows the weight loss and the first derivate (DTG) for all the samples, while the main results are summarized in Table 1. Neat PLA decomposed in a single-step process with a maximum degradation rate (T_max_) at 375 °C, in agreement with values previously reported [27,37].

As expected, the maximum degradation temperature for both plasticized PLA is much smaller than neat PLA, being, however, higher than the temperature shown for the processing of these materials, which is 160 °C. These results demonstrate that both plasticizers can be processed at the same melt processing window of neat PLA avoiding their thermal degradation. The same occurs for TPS, with a T_max_, of about 300 °C. Moreover, from Figure 4, it is possible to note the different stages of the thermal decomposition mechanism of TPS. In particular, the first stage is related to the physical dehydration of water, volatile compounds, and glycerol decomposition while the second stage is related to the chemical dehydration of bonded water and the thermal decomposition of starch [38]. Analyzing the thermal stability of the plasticized PLA/TPS blends, it is easy to note that it is composed of a two-step degradation and that they presented intermediate thermal stability compared with both neat polymers. In particular, the DTG curves show a main peak of degradation related to PLA decomposition, and a small shoulder at lower temperatures related to the TPS thermal decomposition. Regarding the blends, moPLA/TPS showed the highest thermal stability in terms of T_max_, about 340 °C, ten degrees higher than oPLA/TPS T_max_. These results indicate that mOLA8 is able to increase the compatibility of plasticized PLA/TPS blends, as previously observed in the morphology analysis. 

**Figure 5:**
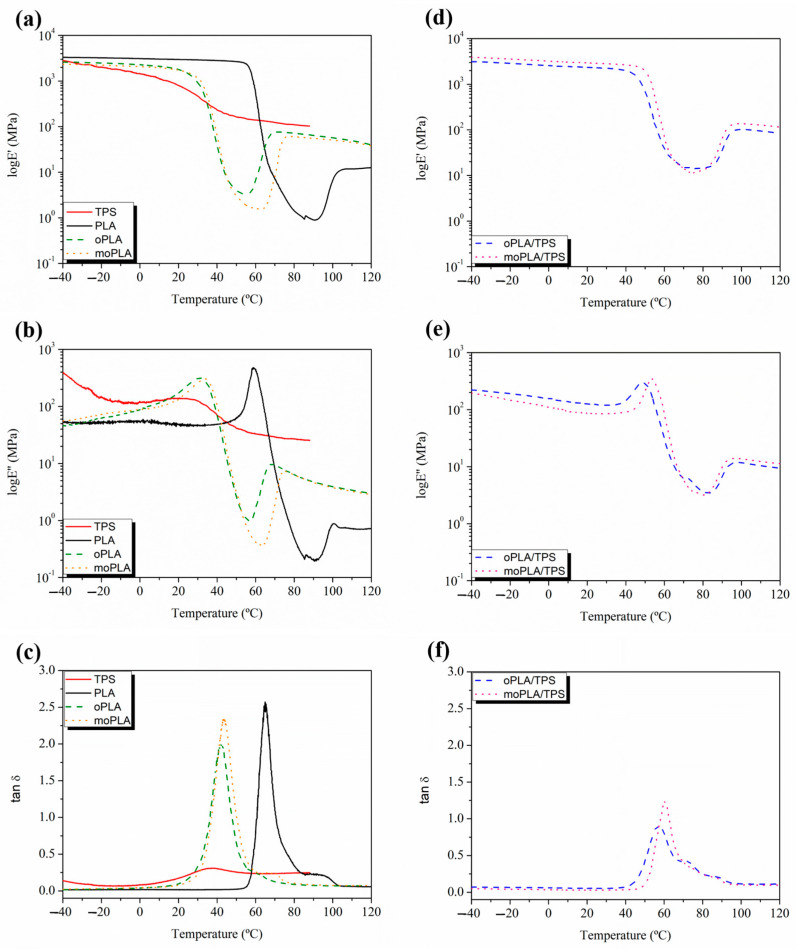
Comparison of the evolution of the storage modulus (E’) (**a**,**d**), loss modulus (E”) (**b**,**e**), and damping factor (tanδ) (**c**,**f**) for all the materials.

Figure 5 shows the DMTA curves for neat PLA and TPS, plasticized PLAs, on the left and their blends, on the right. The variation in the E’ values of neat PLA (Figure 5a) showed a dramatic drop between 55 °C and 80 °C, which is representative of the relaxation process of the PLA chains due to the glass transition temperature. A second transition is observed at around 110 °C that reflects the cold crystallization of PLA. Adding both plasticizers, the E’ curves and consequently their drop, shifted to lower temperatures indicating a decrease in T_g_ and the T_cc_, as previously observed by DSC analysis. Furthermore, the addition of both plasticizers leads to a broadening of the T_g_ peak (Figure 5b,c) indicating a wide range of relaxation times. Moreover, the E’ values decreased with the addition of both plasticizers, suggesting a decrease in rigidity and an increased ductility of the materials. More accurate T_g_ values can be obtained by determining the peak maximum of tanδ and loss modulus (Figure 5b,c). Observing the tanδ curve, neat PLA showed a T_g_ of 65 °C and the plasticized PLA reached the values of 44 °C and 42 °C for moPLA and oPLA, respectively. These results are in agreement with the results obtained by the DSC characterization.

Moreover, according to the literature [39], TPS shows two relaxations due to a phase-separated system. The first one below −40 °C, termed β-relaxation, which is ascribed to a starch-poor phase rich in glycerol–water content while the relaxation at around 25 °C is termed α-relaxation and is ascribed to a starch-rich phase. Both TPS relaxations are highly dependent on the amount of bonded and non-bonded water enclosed in the starch chains, which can act as a plasticizer decreasing their temperatures. 

Regarding the blends based on plasticized PLA and TPS, it is easy to note that the characteristic relaxations of PLA are shifted to higher temperatures compared with those of plasticized PLA. In this case, the plasticizers are acting partly as plasticizer for PLA and as compatibilizer for the blends, interacting with both PLA and TPS. The typical interactions in these systems are hydrogen bonding between the hydroxyl groups from PLA or starch and hydroxyl, as well as oxygen, from the OLA units, which caused the broadening of the PLA glass transition peak, as previously reported [40]. Moreover, the E’ values of the blends at 25 °C significantly increased compared with those of plasticized PLAs, reaching values close to that of neat PLA. 

**Figure 6:**
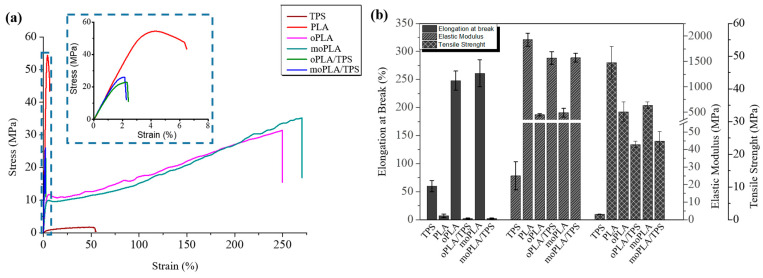
(**a**) Stress vs. strain diagrams for neat PLA and TPS, plasticized PLAs, and their blends with TPS. (**b**) Summary of the mechanical properties.

Mechanical properties of neat PLA, plasticized PLAs, and their blends with 40 wt.% of TPS were measured by tensile test. The tensile mechanical behavior of all the materials can be observed in Figure 6a. When looking at the mechanical properties, plasticizers increase the ductility of the PLA-based materials leading to a higher elongation at break and reducing their stiffness and brittleness. As mentioned before, the mechanism involved in the plasticization process can be described by the interaction of the plasticizer molecules with the PLA chains, reducing the intermolecular forces, which results in an increase in free volume and chain mobility. The results of tensile tests are resumed in Table 1. 

It is easy to notice that plasticized PLA-based materials are more flexible than neat PLA. At the same time, a high decrease in the elastic modulus as well as in the tensile strength is observed compared with neat PLA. Therefore, the use of both plasticizers could lead to plasticized PLA-based materials with better performance for applications where high ductility is required compared with other plasticizers [27]. Therefore, both plasticizers showed similar effects on tensile properties giving comparable values of tensile strength, elastic modulus, and elongation at break. 

**Figure 7:**
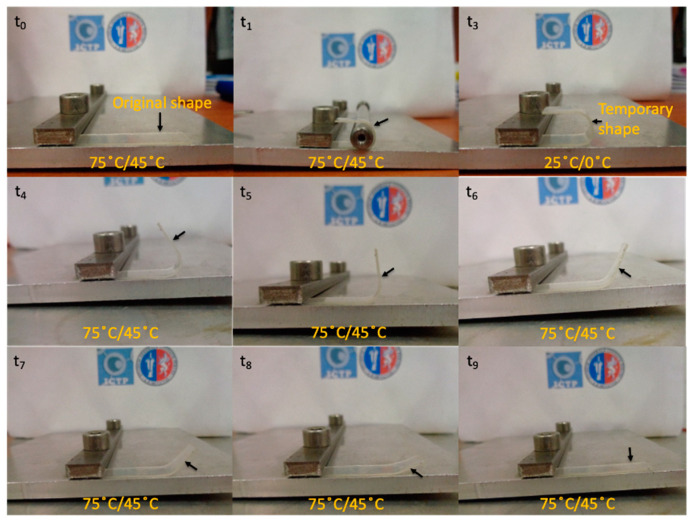
Visual appearance of the thermally activated shape memory process for the sample studied indicating the selected T_trans_ (75 °C for PLA and 45 °C for the rest of materials) as well as the selected T_fix_, in particular 25 °C for PLA and 0 °C for the rest of materials. The arrows indicate the sample during its deformation/recuperation stage.

Moreover, when the plasticized PLA-based materials are blended with TPS, they are able to considerably increase the elastic modulus, slightly decrease their tensile strength, and dramatically drop down the elongation at break reaching values similar to that of neat PLA. Finally, the effect of the PLA plasticization and the compatibilization of its blends with TPS on the thermally-activated shape memory properties were studied in order to evaluate if these materials can overcome the PLA limitation and open up its potential application in biomedical field [31]. Therefore, knowing the morphology of the materials as well as their mechanical and thermal properties, and considering that in general a T_trans_ ≈ T_g_ + 15 °C [31,32] is shown to study the thermally-activated shape memory response, we chose two different transition temperatures, one for neat PLA and one for the plasticized systems, closer to the human body temperature. In particular, the shape memory of neat PLA was thermally-activated at 75 °C while that of plasticized PLA-based materials and their blends with TPS was activated at 45 °C for comparison. A visualization test, Figure 7, of the thermally-activated shape memory properties of neat PLA was done by bending the sample at the T_trans_ in the temporary shape then fixed at room temperature and recovered at the same temperature. The mechanism proposed involved a permanent network, composed of PLA chains physically entangled or linked thanks to the PLA crystals, able to memorize the permanent shape and store the driving energy needed to recover it. On the other hand, the switching phase, characterized by the T_trans_, is formed by the amorphous PLA chains which can be frozen below their T_g_ in order to fix the temporary shape and reheated above their T_g_ to gain high mobility and activate the recovery of the permanent shape.

The thermo-mechanical cycles were performed at 50% elongation. The programming step was designed with a uniaxial stretching at the selected T_trans_ (75 °C for PLA and 45 °C for the rest of materials), followed by a fast quenching of the stretched state at the T_fix_, in particular 25 °C for PLA and 0 °C for the rest of materials. For PLA, it is not necessary bring down the fix temperature to 0 °C. However, for plasticized systems, taking into account that the transition temperature is only 45 °C, we have to choose a fix temperature lower than 25 °C in order to fix the temporary shape, choosing a difference of about 50 °C between both temperatures, in both cases, therefore a T_fix_ of 0 °C for the plasticized samples.

**Figure 8:**
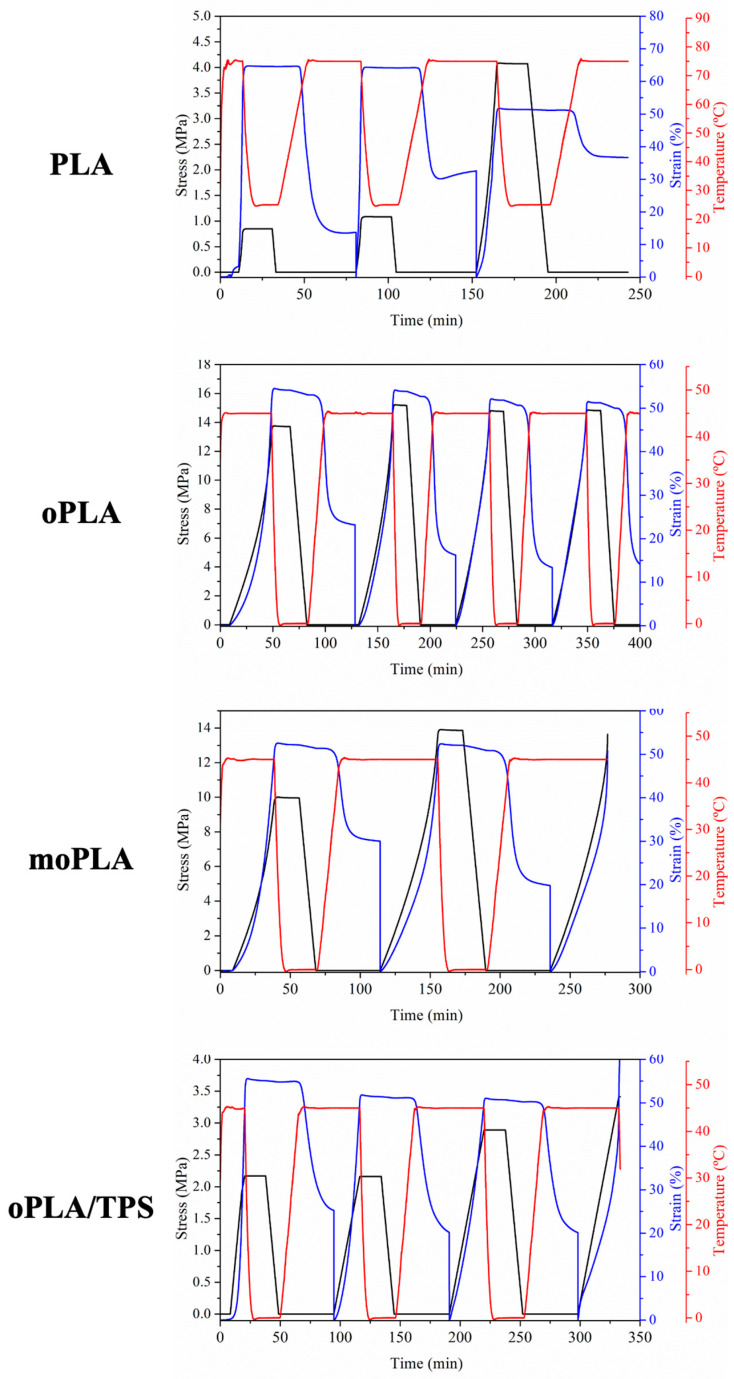
Evolution of stress, strain, and temperature in function of time and 3D stress–strain–temperature for all the materials.

The stretched state was maintained after quenching and subsequent removal of the stress at the same temperature. Finally, the recovery of the permanent shape was activated at the T_trans_. Figure 8 presents the evolution of strain, stress, and temperature in function of time during the dual-shape memory programming step for all the materials. The results related to the sample moPLA/TPS at 45 °C are not shown because it was not possible to perform the thermo-mechanical cycle due to the breaking of the sample. 

It is worth noting that PLA showed an increase in the applied stress increasing the number of cycles, probably due to the induced crystallization as a consequence of the annealing process triggered at the T_trans_ during the recovery stage of the thermo-mechanical cycle. The effect of the annealing process was already reported in the literature for PLA/PCL blends demonstrating that the increase in the degree of crystallinity can increase the mechanical properties [32]. The effect of PLA annealing is also visible in the values of R_r_ obtained for PLA by the thermo-mechanical cycles. In Table 2, the results of the shape memory properties characterization, in terms of R_r_ and R_f_, are summarized for all the samples. As it is easy to notice, PLA showed an excellent ability to fix the temporary shape, showing maximum values as high as 100%. On the other hand, the R_r_ values of PLA decreased, increasing the number of cycles and reaching minimum values of 28%. As mentioned above, this was probably due to the induced crystallization at the T_trans_ used, which is largely above the T_g_ of PLA. Thus, the mobility of the chains is increased and during the recovery step they might have the time to organize themselves in ordered crystals avoiding complete recovery. This behavior is due to the lack of stiffness of the permanent network that compose the PLA sample which is characterized mainly by physical entanglement that at the T_trans_ might not store all the driving energy needed to recover the permanent shape in the following step. Recently, Leones et al. [28] reported the shape memory ability of neat electrospun PLA fibers thermally-activated at 60 °C showing excellent values of R_r_ and R_f_. In their case, a part to adapt the T_trans_ used to be equal to the T_g_ of neat PLA, avoiding in this way the relaxation of the permanent network, and the neat PLA electrospun fibers showed a 5% of degree of crystallinity, being enough to increase the stiffness of the physical network of the sample at the T_trans_. 

Therefore, in our systems, oPLA showed excellent values of R_f_ and high values of R_r_. This improvement is due to the fact that the permanent network of this sample is composed by physical entanglement together with crystals that increase the stiffness of the network that has to store the stretching energy during the programming step. 

Looking at the shape memory ability of moPLA, we can easily notice that it was not possible to complete more than 2 consecutive cycles with the same sample. The applied stress needed to reach the elongation of 50% at the T_trans_ was higher compared with oPLA. Evaluating the cycles obtained, it was possible to observe that the sample showed a higher R_r_ value in the second cycle while excellent R_f_ values were obtained for both cycles. Moreover, oPLA/TPS showed excellent R_f_ values and good R_r_ values. Compared with its counterpart without TPS, we can state that the thermally-activated shape memory ability depending on the PLA-based phase was almost totally preserved even while using 40 wt.% of TPS. Finally, as we mentioned above, it was not possible to perform the thermo-mechanical cycles with the compatibilized blend, moPLA/TPS. The high degree of crystallinity of the blend (20%) probably designed a stiff and brittle permanent network to be stretched at the T_trans_ used, leading to the breaking of the sample. We can conclude that plasticized PLA/TPS blends show good thermally-activated shape memory response in the case of oPLA/TPS but the improvement of their compatibility in the moPLA/TPS leads to the loss of this property. 

## 6. Conclusions

Biodegradable blends based on plasticized PLA and thermoplastic starch have been obtained and characterized. It is very interesting to use the same additive able to act as both plasticizer and compatibilizer, decreasing the glass transition temperature of PLA to a temperature close to the physiological one, obtaining a material suitable for potential biomedical applications. However, the thermally-activated shape memory results show that it is very important to make a balance between plasticizer and compatibilizer, considering the need of two well-established phases to obtain shape memory response, the fix one and the shift one. The blend moPLA/TPS does not show thermally-activated shape memory response, while oPLA/TPS is able to present thermally-activate shape memory capability at 45 °C and 50% deformation. Thus, OLA8t plasticizing effect leads to different improvements such as the decrease in the system T_g_ to a temperature close to the human body temperature and the consequent use of a T_trans_ suitable for biomedical application. On the other hand, the plasticizer addition increases the degree of crystallinity of the sample, improving the properties of the permanent network which is essential to reach good shape memory response. Finally, oPLA/TPS presents optimal values of R_f_ and R_r_ at a deformation of 50%. Therefore, this analysis opens the way to a deeper study on the shape memory response of this biodegradable and biocompatible system for further biomedical application. 

## Figures and Tables

**Table 1:** Mechanical properties of all the samples.

Sample	E (MPa)	Ts (MPa)	ε (%)
PLA	1930 ± 130 ^a^	48 ± 5 ^a^	7 ± 3 ^a^
TPS	25 ± 8 ^b^	1.7 ± 0.1 ^b^	60 ± 10 ^b^
oPLA	450 ± 20 ^c^	33 ± 3 ^c^	248 ± 17 ^c^
moPLA	490 ± 88 ^c^	35 ± 1 ^c^	261 ± 24 ^c^
oPLA/TPS	1568 ± 126 ^d^	23 ± 1 ^d^	2 ± 1 ^a^
moPLA/TPS	1573 ± 87 ^d^	24 ± 3 ^d^	2 ± 1 ^a^
F ratio	5.30	3.82	4.65
*p*-Value	0.00847 *	0.02974 *	0.01588 *

Different letters in the column indicate significant differences according to Tukey’s test (*p* < 0.05). * Values are significant at *p* < 0.05.

**Table 2:** R_r_ and R_f_ values for all the sample tested by the thermo-mechanical cycles.

Sample	T_trans_ (°C)	T_fix_	R_f_ (%) Cycle				R_r_ (%) Cycle			
			1st	2nd	3rd	4th	1st	2nd	3rd	4th
PLA	75	25	100	100	100	-	79	53	28	-
oPLA	45	0	97	98	97	97	57	70	74	75
moPLA	45	0	98	97	-	-	43	62	-	-
oPLA/TPS	45	0	99	99	99	-	55	61	61	-

## Data Availability

The data that support the findings of this study are available from the corresponding author upon reasonable request.
